# Human papillomavirus infection and cervical intraepithelial neoplasia progression are associated with increased vaginal microbiome diversity in a Chinese cohort

**DOI:** 10.1186/s12879-020-05324-9

**Published:** 2020-08-26

**Authors:** Yulian Chen, Xingdi Qiu, Wenjing Wang, Dong Li, Anyue Wu, Zubei Hong, Wen Di, Lihua Qiu

**Affiliations:** 1 Department of Gynecology and Obstetrics, Ren ji Hospital, School of Medicine, Shanghai Jiao Tong University, Shanghai, China; 2 Shanghai Key Laboratory of Gynecologic Oncology, Ren ji Hospital, School of Medicine, Shanghai Jiao Tong University, Shanghai, China; 3 State Key Laboratory of Oncogenes and Related Genes, Shanghai Cancer Institute, Ren ji Hospital, School of Medicine, Shanghai Jiao Tong University, Shanghai, China

**Keywords:** Vaginal microbiome, Human papillomavirus, Cervical intraepithelial neoplasia, Cervical cancer

## Abstract

**Background:**

In this study, the association between human papillomavirus (HPV) infection and related cervical intraepithelial neoplasia (CIN) or cervical cancer and vaginal microbiome was evaluated in Chinese cohorts.

**Methods:**

The vaginal bacterial composition of five groups, HPV-infected women without CINs (HPV, ***n*** = 78), women with low-grade squamous intraepithelial lesions (LSIL, ***n*** = 51), women with high-grade squamous intraepithelial lesions (HSIL, ***n*** = 23), women with invasive cervical cancer (Cancer, ***n*** = 9) and healthy women without HPV infection (Normal, ***n*** = 68), was characterized by deep sequencing of barcoded 16S rRNA gene fragments (V3**–**4) using Illumina MiSeq.

**Results:**

HPV infection increased vaginal bacterial richness and diversity regardless of the status of CINs. The vaginal bacterial richness and diversity were further augmented in women with cervical cancer. *Lactobacillus* was the most abundant genus in all groups. HPV infection had a negative influence on the abundances of *Lactobacillus*, *Gardnerella* and *Atopobium*. Accordingly, HPV infection increased the relative abundance of *Prevotella*, *Bacillus*, *Anaerococcus*, *Sneathia*, *Megasphaera*, *Streptococcus* and *Anaerococcus*. The increased proportions of *Bacillus*, *Anaerococcus* and the reduced abundance of *Gradnerella vaginalis* were probably related with the progression of CINs severity. HPV infection without CINs or cancerous lesions was strongly associated with *Megasphaera*. The most abundant bacterium in the LSIL group was *Prevotella amnii*. However, *Prevotella timonensis*, *Shuttleworthia* and *Streptococcaceae* at the family level were three taxa related to HSIL. Furthermore, more taxa were associated with the Cancer group including *Bacillus*, *Sneathia*, *Acidovorax*, *Oceanobacillus profundus*, *Fusobacterium*, *Veillonellaceae* at the family level, *Anaerococcus* and *Porphyromonas uenonis*. Samples in the Normal group were mostly assigned to CST III. HPV infection converted the vaginal bacterial community structure from CST III to CST IV. Furthermore, the proportions of CST IV were gradually augmented with the progression of the severity of CINs.

**Conclusions:**

This work interpreted the differential vaginal bacteria under HPV infection and various precancerous or cancerous lesions in a Chinese cohort. We distinguished the specific microbes and the vaginal bacterial structure that were related with the progression of CINs severity in Chinese women.

## Background

Human papillomavirus (HPV) is regarded as one of the most common sexually transmitted agents in cervical intraepithelial neoplasia (CIN) and cervical cancer [1]. High-risk subtypes of HPV contribute to 99% of cervical neoplasia [1]. However, it is known that high-risk HPV infection is necessary but not sufficient for the development of CINs or cervical cancer [2–4]. Many other events, such as multiple sexual partners, early initiation of sexual activity and co-infection with other sexually transmitted infections, have been associated with higher risk of HPV infection in the genital tract [5–7].

In the female genital tract, a healthy vaginal status is commonly associated with low microbial diversity and prevalence of *Lactobacillus*. *Lactobacillus* prevents colonization of other bacterial pathogens through production of lactic acid, hydrogen peroxide (H_2_O_2_) and bacteriocin in the vagina, and therefore keeps integrity of mucosal barriers against virus and opportunistic bacteria [4, 8, 9]. The vaginal microbial profile of women could by classified into five community state types (CSTs) by hierarchical taxonomic clustering, in which CSTs I, II III and V are predominated by *L. crispatus*, *L. iners*, *L. jensenii* and *L. gasseri* respectively, while CST IV is depleted of *Lactobacillus* and enriched with anaerobic bacteria like *Gardnerella*, *Megasphera*, *Sneathia*, *Prevotella,* etc. [10]. Commensal vaginal *Lactobacillus* species are thought to defend against many pathogens, such as *Candida* infection [11, 12], sexually transmitted diseases [13], urinary system infections [14, 15] and human immunodeficiency virus (HIV) infection [16]. However, *L. iners* has many properties different from other *Lactobacillus spp.*, for example unable to produce H_2_O_2,_ and it often predominates in the presence of HPV infection [4, 17, 18] and CIN [18, 19].

Bacterial vaginosis (BV) is a cluster of microbial disorders characterized by a decrease in *Lactobacillus* and their replacement by high concentrations of other anaerobic bacteria, with a microbial community structure in accordance with CST IV [9]. BV is associated with a higher risk of miscarriage, preterm premature rupture of membranes and a higher susceptibility to sexually transmitted infections, as HPV infection [20–22]. Some studies to date have reported that vaginal microbiome (VM) plays an important role in the persistence of the HPV infection and the subsequent development of cervical precancerous or cancerous lesions [2, 4, 8, 17–19, 23–28]. Increasing VM diversity is associated with advancing CINs severity and viral persistence [26]. The potential mechanisms could be linked to less production of protective lactic acid, H_2_O_2_ and bactriocin by *Lactobacillus*, disruption of mucosal integrity which may aid viral entry, higher levels of oxidative stress induced by dybiosis [8]. Particular species like *Sneathia spp.* have a probable pathological role in HPV acquisition and persistence through cellular targets such as expression of immunosuppressive cytokines [29]. Therefore, it is considerable to take vaginal microbiome as a promising marker not only for HPV infection but also for cervical precancerous lesions.

Nevertheless, the vaginal communities could be influenced by many other factors, including ethnicity, personal hygiene, sexual behaviors and hormonal levels [10, 30]. Ethnicity is key to shape vaginal bacterial communities [4, 10, 31]. Caucasian and Asian women display a significantly greater prevalence of *Lactobacillus* in the vagina compared to Hispanic and Black women [8, 10, 31]. To our knowledge, data with regard to the vaginal bacterial composition of Chinese populations are inadequate [8, 18, 32]. The analysis, which is performed in a large cohort of women living in a different country and with supposed different hygiene habits [10], is helpful to reinforce the underlying associations. Furthermore, there are few studies about the association between VM and HPV infection and related CIN diseases in Chinese cohorts using high throughput sequencing method [8, 18, 32].

Hence, the objective of this research is to study the role of VM on the HPV infection and the progression of CIN diseases in Chinese populations. We try to identify the microbiological markers related with HPV infection and CINs severity in these cohorts.

## Methods

### Study population and sample collection

We included 229 non-pregnant women, 25–69 years of age, who attended gynecological clinics at the Department of Gynecology, Renji Hospital of Shanghai, Jiao Tong University School of Medicine, between May 2016 and November 2016. Non-pregnant women were included irrespective of their phase in their cycle (except for the menstrual period), parity, personal hygiene, smoking habits. No previous medical histories of CIN diseases or cervical cancer and other serious medical problems, such as hepatitis B/C, diabetes, autoimmune diseases, sexually transmitted diseases (chlamydia, gonorrhea, trichomoniasis, genital herpes), HIV or other malignant tumors, were declared. Participants who had vaginal intercourse or vaginal douching within last 3 days of sampling, abnormal metrorrhagia in the previous weeks, or used probiotics, antibiotics or immunosuppressive drugs in the preceding 14 days were excluded.

HPV genotyping test and ThinPrep cytology test (TCT) were carried out in all enrolled patients, using a commercial HPV genotyping kit as previously described [33]. TCT results were interpreted on the basis of Bethesda System criteria [34]. Women with HPV positive and/or TCT ≥ ASCUS accepted the biopsy under the colposcopy examination by two gynecologists. Based on their histopathology, all participants were assigned into five groups as follow (Fig. S1): HPV-infected women without CINs (HPV, *n* = 78), women with low-grade squamous intraepithelial lesions (LSIL, *n* = 51), women with high-grade squamous intraepithelial lesions (HSIL, *n* = 23), women with invasive cervical cancer (Cancer, *n* = 9) and healthy women without HPV infection (Normal, *n* = 68).

Sterile swab samples for 16S rRNA sequencing were taken from the lateral and posterior fornix using a sterile speculum as previously described [33].

### Total bacterial genomic DNA extraction and MiSeq sequencing

Primers 338F (ACTCCTACGGGAGGCAGCA) and 806R (GGACTACHVGGGTWTCTAAT) were used to amplify the V3–4 hypervariable fragments of the 16S rRNA gene by PCR as previously described [33]. All sequencing was conducted using the Illumina MiSeq platform at Majorbio Biopharm Technology Company (Shanghai).

### Sequence analysis

Sequence reads were quality checked by Trimmomatic [35]. OTUs were generated by QIIME and taxonomies were classified using the Ribosomal Database Project (RDP) classifier script (version 2.2) as previously described [33].

As previously described [33], alpha (Chao and Shannon index) and beta indices (unweighted UniFrac distances in Principal coordinates analysis (PCoA)) were calculated by mothur (version v.1.30.1) [36] and vegan package in R [37]. We compared the differences of alpha and beta estimators between two groups by Student’s t-test and ANOSIM test respectively. Heat maps of relative abundance for different taxa were generated using R. The relative abundances of different taxa at different levels between the five groups were calculated by nonparametric Wilcoxon test. Linear discriminant analysis (LDA) effect size (LEfSe) algorithm [38] was used to characterize the potential microbial markers with specific disease phenotypes. Hierarchical clustering analysis was used to classify different vaginal community state types (CSTs) as previously published [31, 39]. Q-values (*p*-value adjusted by false discovery rate (FDR)) and *p*-values < 0.05 were considered significant.

## Results

### Characteristics of the study population

The average ages of each group (HPV, LSIL, HSIL, Cancer, Normal) were 47.78 ± 9.63, 46.00 ± 10.19, 43.70 ± 10.74, 56.11 ± 9.02 and 43.00 ± 8.69 years, respectively. The proportions of 16/18 HPV subtypes in HPV positive groups were as follow: HPV (13/78), LSIL (10/51), HSIL (6/23), Cancer (4/9) (Table S1). The demographic characteristics of each group are presented in the supplementary data (Table S2).

### Sequencing results

After filtering low-quality reads, 6,585,141 assembled clean reads were obtained from 229 samples, with an average number of reads per sample of 28,755.64 ± 5389.65 and a mean read length of 444.90 ± 5.89 bp. For normalization, the reads in each sample were randomly subsampled to the lowest number of 20,098 in sample 318_LCQ (HPV group). After removing singletons (the OTUs contained less than 2 reads), 1878 OTUs were identified, ranging from 10 OTUs in sample 4391 (Normal group) to 782 OTUs in sample 152_GPZ (HPV group) (Table S3). The average number of OTUs in HPV-negative group (Normal group: 46) was lower than that in HPV-positive groups (HSIL group: 116; LSIL group: 145; HPV group: 157). We found more OTUs in the samples of Cancer group (Mean = 256.70 ± 174.78), ranging from 51 to 599 (Table S3).

### Vaginal microbiota richness and diversity

**Fig. 1:**
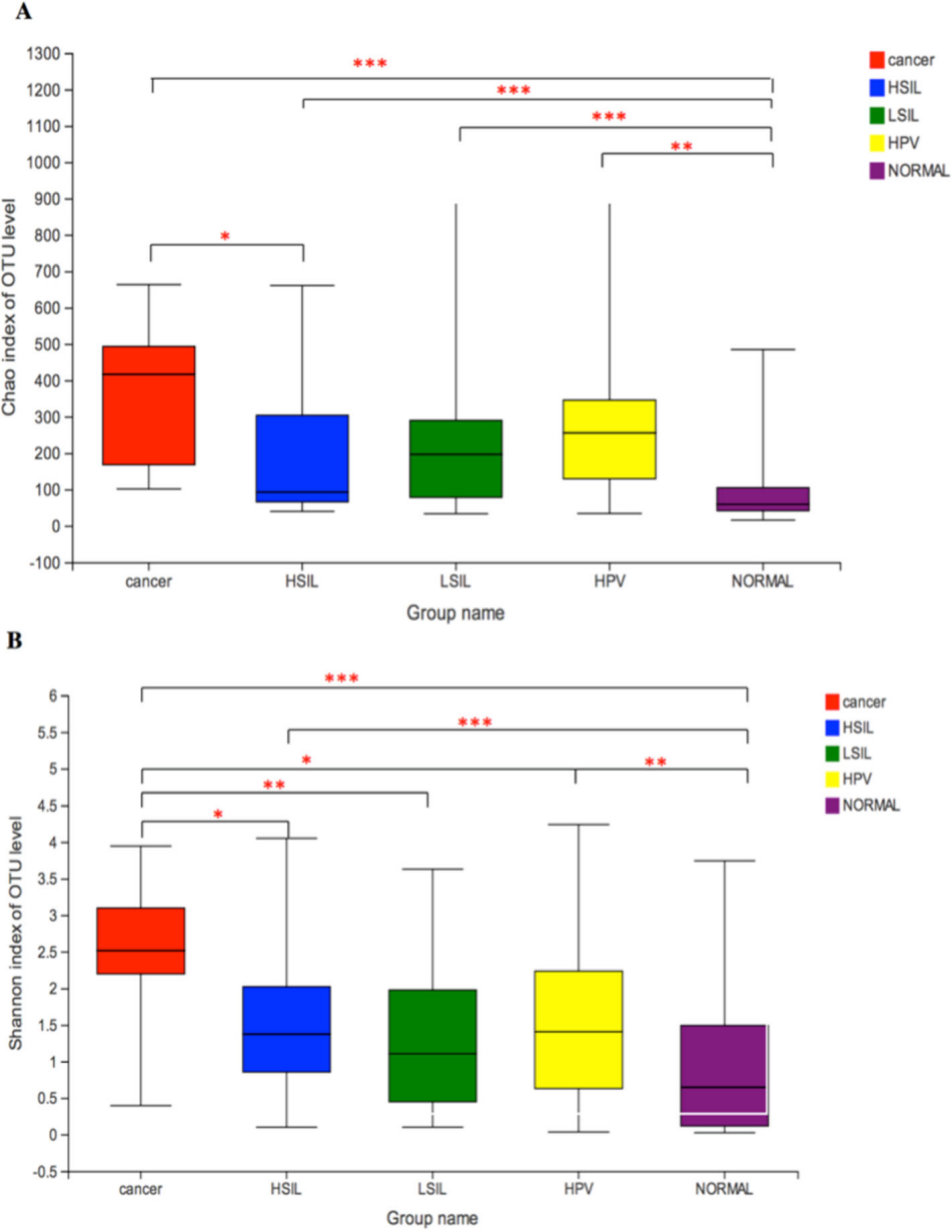
Vaginal bacterial richness and diversity in five groups. **a**: Chao index; **b**: Shannon index; Student’s t-test was used to compare differences between two groups; data are presented as the mean ± SD; ^***^: q ≤ 0.001; ^**^: 0.001 < q < 0.01; *: q < 0.05

At the OTU level, microbial richness and diversity were estimated using Chao and Shannon indices, respectively, as shown in Fig. 1. These two indices revealed that HPV infection increased vaginal bacterial richness and diversity. The means of Chao and Shannon indices were much lower in groups HPV-negative (Normal group: 84.02 ± 73.88 and 0.94 ± 0.95, respectively) than in groups HPV-positive (HPV group: 272.26 ± 191.62 and 1.49 ± 1.01, q < 0.01; LSIL group: 231.84 ± 195.39, q < 0.001 and 1.30 ± 0.93, q = 0.052; HSIL group: 185.74 ± 162.62, q < 0.001 and 1.51 ± 0.98, q = 0.02; Cancer group: 367.76 ± 208.63 and 2.47 ± 0.98, q < 0.001, respectively). The means of Chao and Shannon indices were the highest in group Cancer and were significantly higher than in groups HSIL (Chao: 367.76 ± 208.63 vs. 185.74 ± 162.62, q = 0.02, Shannon: 2.47 ± 0.98 vs. 1.51 ± 0.98, q = 0.02), LSIL (Shannon: 2.47 ± 0.98 vs. 1.30 ± 0.93, q = 0.003) and HPV (Shannon: 2.47 ± 0.98 vs. 1.49 ± 1.01, q = 0.02). However, there were no differences found among groups HPV, LSIL and HSIL.

### Vaginal bacterial structure and beta-diversity in different groups

**Fig. 2:**
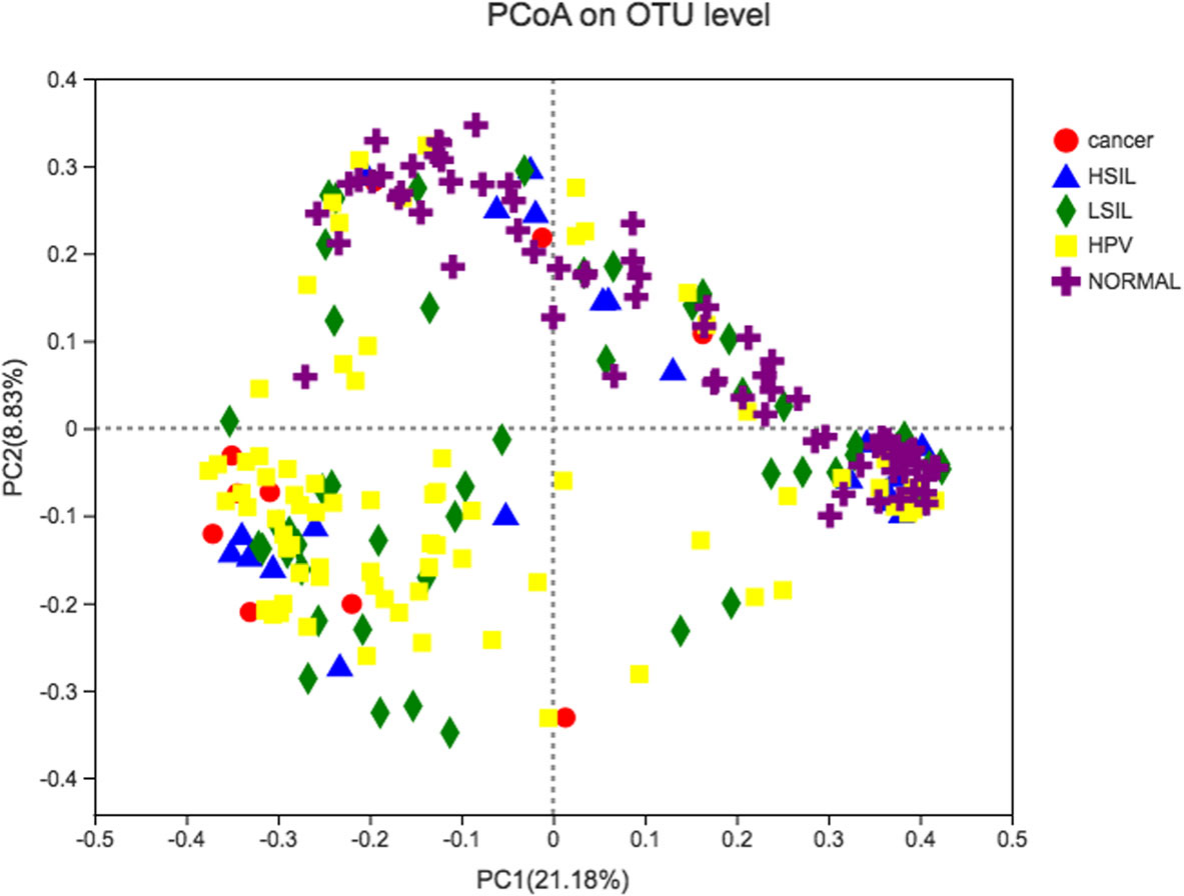
Unweighted UniFrac principal coordinates analysis (PCoA) plot comparing sample distribution for the different groups

In PCoA, the first two principal components explained 21.18 and 8.83%, respectively, of the variance along the first and second axes, with the Cancer, HSIL, LSIL and HPV samples visually separated from the Normal sample (Fig. 2). Comparison between two groups based on the ANOSIM test revealed that the bacterial structure of groups Cancer, LSIL and HPV were significantly different from that of group Normal (Table S4). Meanwhile, the bacterial structure of groups HSIL and LSIL were also different from that of group HPV (Table S4).

### Taxonomy of the vaginal microbiota in different groups

**Fig. 3:**
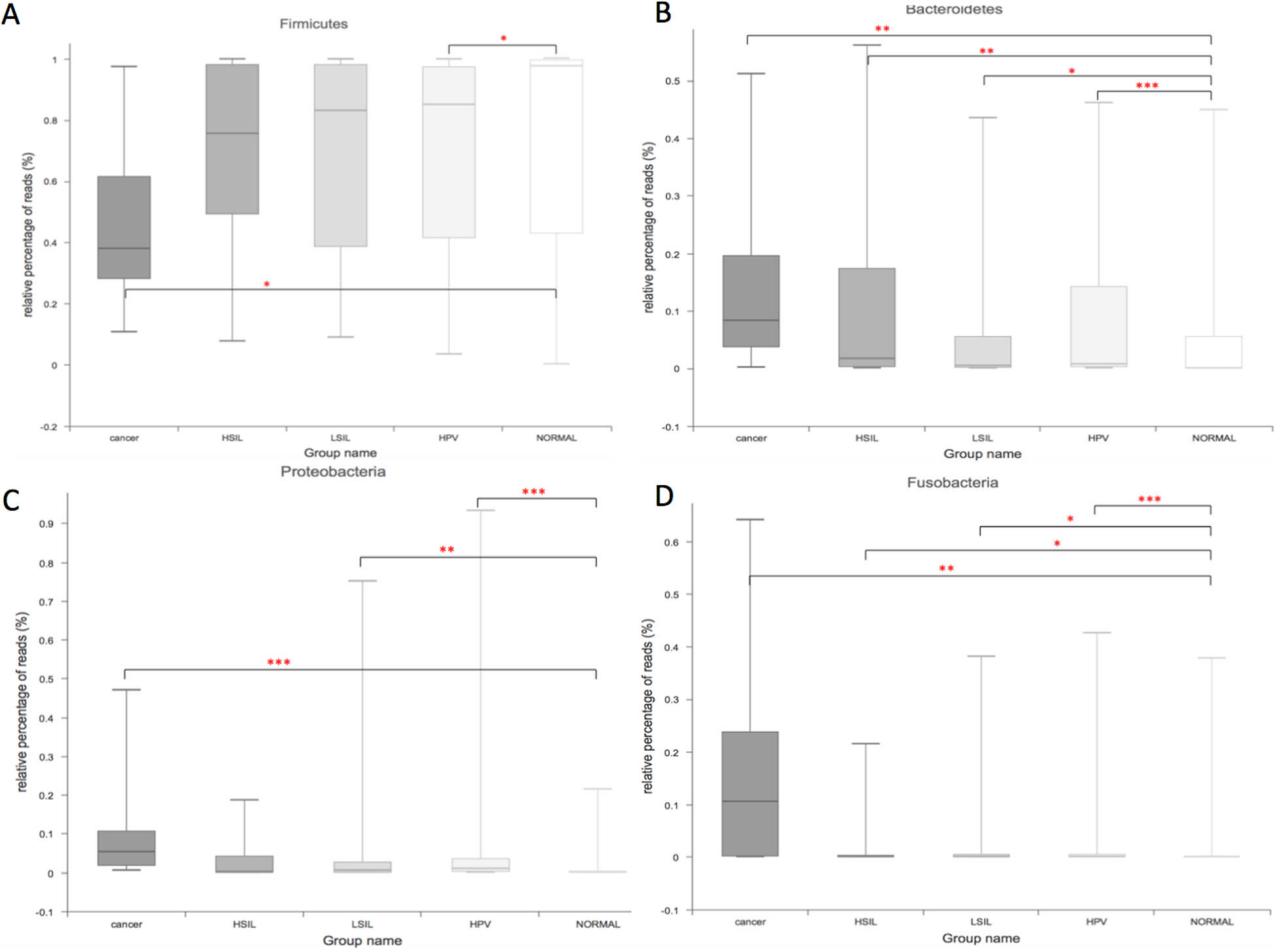
Relative abundance counts of *Firmicutes* (**a**), *Bacteroidetes* (**b**), *Proteobacteria* (**c**) and *Fusobacteria* (**d**), which were found to be the most abundant phyla across all samples. The Wilcoxon test was used to compare differences in the abundance of each phylum between two groups. ^***^: q ≤ 0.001; ^**^: 0.001 < q < 0.01; *: q < 0.05

Overall, 37 bacterial phyla were recovered across all samples (Fig. S2), and All of the samples were dominated by *Firmicutes*, *Actinobacteria*, *Bacteroidetes*, *Fusobacteria* and *Proteobacteria* (Fig. S2). *Firmicutes* was the most abundant phylum, accounting for 73.15, 68.91, 69.70, 70.43 and 47.47% of the Normal, HPV, LSIL, HSIL and Cancer groups, respectively (Fig. 3a). HPV infection tended to lower the proportion of *Firmicutes* (Cancer < Normal, q < 0.05; HPV < Normal, q < 0.05). However, there were no differences among other HPV positive groups. Accordingly, the proportions of *Bacteroidetes*, *Fusobacteria* and *Proteobacteria* were significantly increased in the HPV positive groups as compared to the Normal group (*Bacacteroidetes*: Cancer 15.72% > HSIL 13.1% > HPV 8.28% > Normal 6.94%, Fig. 3b; *Fusobacteria*: Cancer 15.84% > LSIL 3.04% > HSIL 2.62% > HPV 2.64% > Normal 2.54%, Fig. 3d; *Proteobacteria*: Cancer 10.39% > HPV 5.36% > LSIL 3.59% > Normal 1.18%, Fig. 3c). There were no differences among the different groups with regard to the proportion of *Actinobacteria*.

**Fig. 4:**
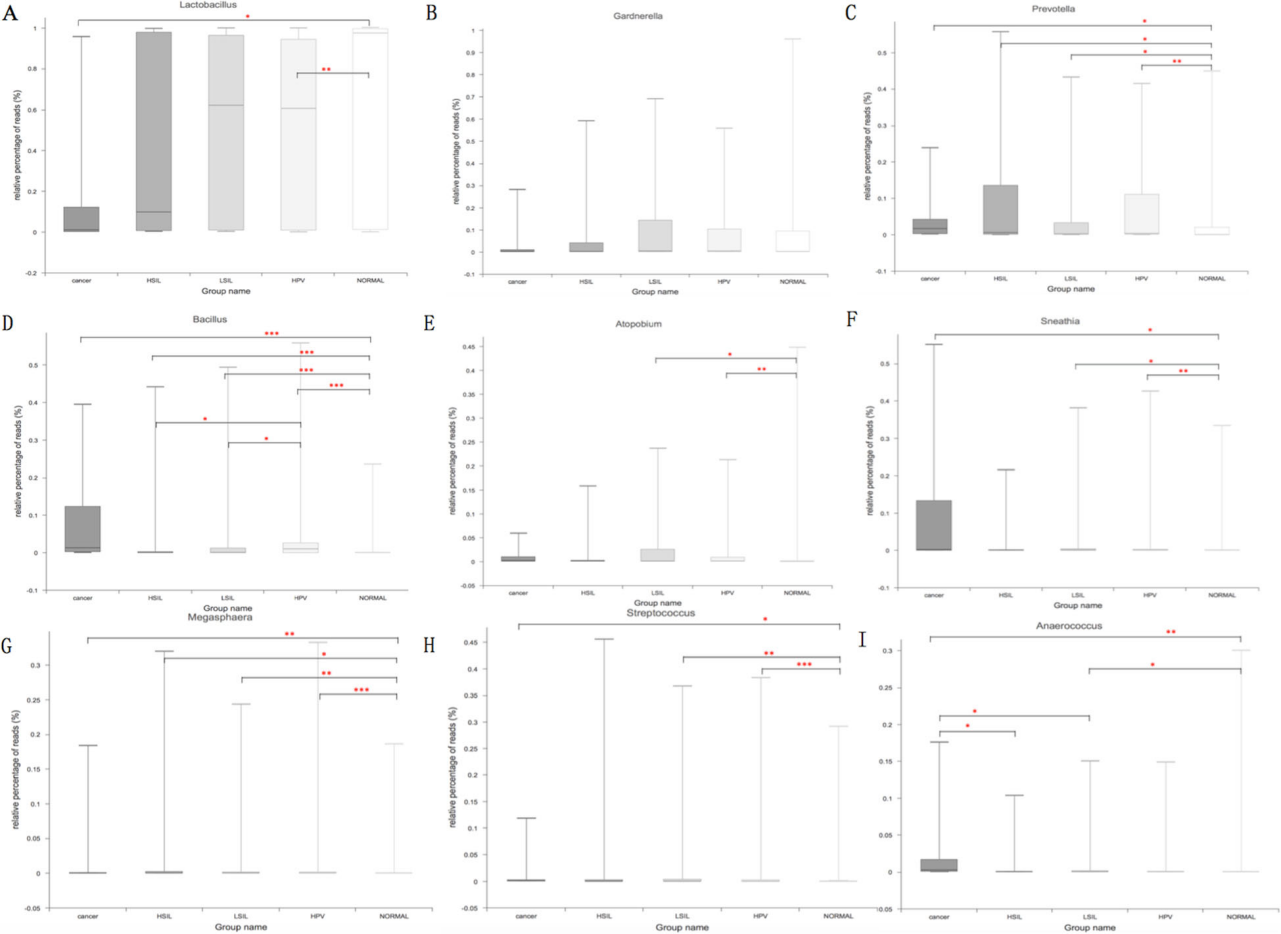
Relative abundance counts of *Lactobacillus* (**a**), *Gardnerella* (**b**), *Prevotella* (**c**), *Bacillius* (**d**), *Atopobium* (**e**), *Sneathia* (**f**), *Megasphaera* (**g**), *Streptococcus* (**h**) and *Anaerococcus* (**i**), which were found to be the most abundant genera across all samples. The Wilcoxon test was used to compare differences in the abundance of each phylum between two groups. ^***^: q ≤ 0.001; ^**^: 0.001 < q < 0.01; *: q < 0.05

At the genus level, a total of 749 taxa were found across all samples (Fig. S3), with *Lactobacillus* being the most dominant genus overall. The proportion of *Lactobacillus* was reduced in the HPV positive groups (Cancer: 19.72%, q < 0.05; HSIL: 42.99%, q = 0.098; LSIL: 53.71%, q = 0.051; HPV: 49.20%, q < 0.01, respectively) compared to the Normal group (64.93%) (Fig. 4**a**). However, there were no significant differences among the groups HPV, LSIL, HSIL and Cancer with regard to the relative abundance of *Lactobacillus*. There were no differences among the different groups with regard to the abundance of *Gardnerella* (Fig. 4**b**). In addition, HPV infection had also a negative effect on the abundance of *Atopobium*, which sharply decreased from 3.12% in the Normal group to 2.07% in the HPV group (q < 0.01), 2.72% in the LSIL group (q < 0.05) (Fig. 4**e**). Accordingly, HPV infection increased the relative abundance of *Prevotella* (Normal: 5.91% < HPV: 7.18%, q < 0.05, Fig. 4**c**), *Bacillus* (Normal: 0.34% < LSIL: 3.30% < HPV: 5.77% < HSIL: 5.87% < Cancer: 9.18%, q < 0.001, Fig. 4**d**), *Sneathia* (Normal: 2.39% < HPV: 2.61% < LSIL: 3.02% < Cancer: 10.03%, q < 0.05, Fig. 4**f**), *Megasphaera* (Normal: 1.01% < LSIL: 2.13% < Cancer: 2.30% < HSIL: 2.57% < HPV: 9.18%, q < 0.05, Fig. 4**g**), *Streptococcus* (Normal: 1.03% < Cancer: 1.29% < LSIL: 1.35% < HPV: 1.93%, q < 0.05, Fig. 4**h**) and *Anaerococcus* (Normal: 1.22% < Cancer: 3.83%, q < 0.05, Fig. 4**i**). The precancerous lesions increased the proportion of *Bacillus*, with a greater bacterial abundance in LSIL and HSIL groups than in HPV group (q < 0.05) (Fig. 4**d**). A higher proportion of *Anaerococcus* was also found in the group Cancer compared to the precancerous groups (HPV, LSIL and HSIL, q < 0.05) (Fig. 4**i**). Two taxa, *Bacillus* and *Anaerococcus*, were probably related with the progression of CINs severity.

### Identification of vaginal microbiological markers in different groups

**Fig. 5:**
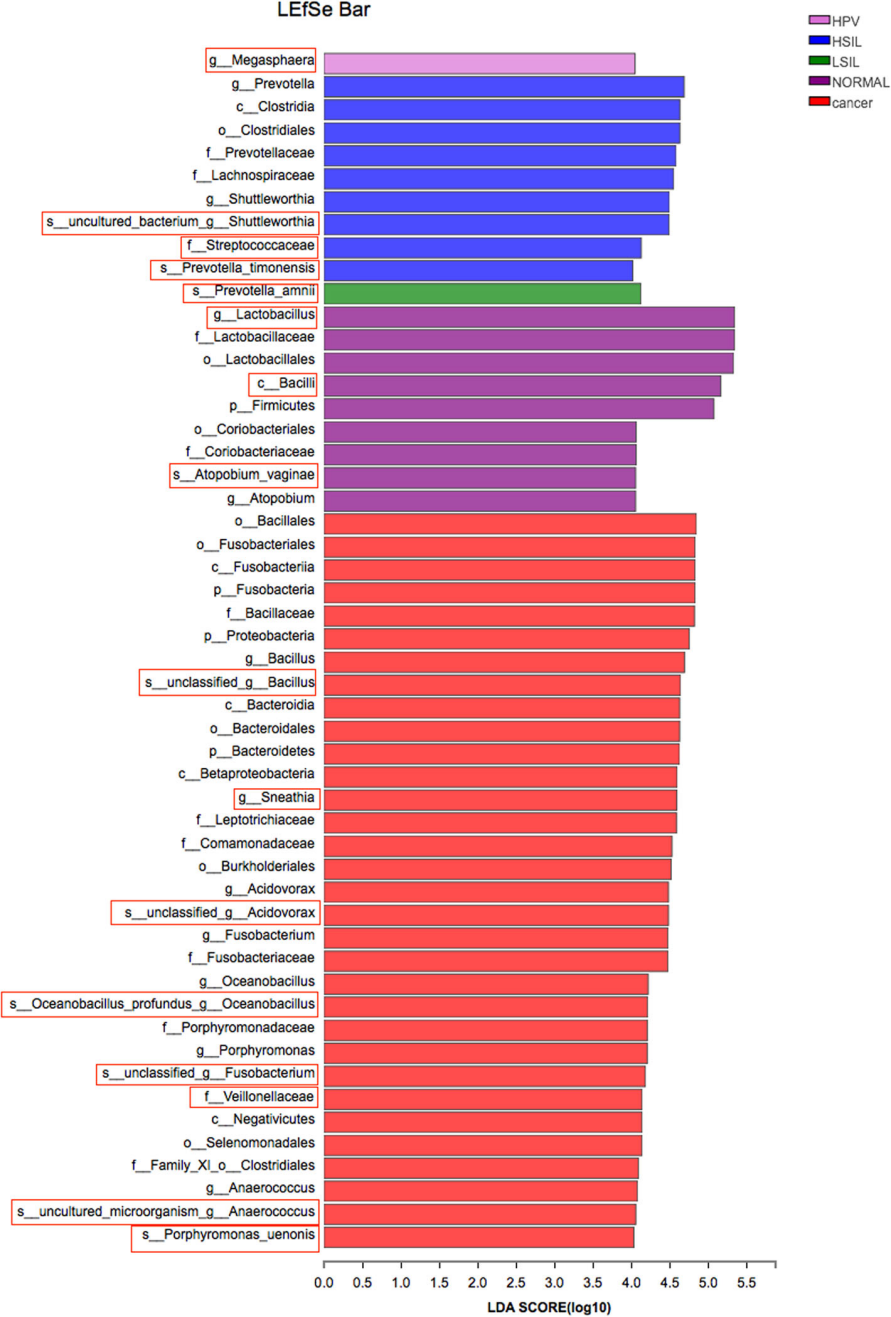
The unique taxa and microbiomarkers for different groups. Shown is a histogram of LDA scores computed for features differentially abundant in the five groups

LEfSe modeling was employed to identify microbiological markers related to HPV infection and CINs severity (Fig. 5). The threshold for the logarithmic LDA model score for discriminative features in this study was 4.0 (*p* < 0.05). The most abundant genus in the Normal group was *Lactobacillus.* In addition, other two taxa were also more abundant in the Normal group, *Bacilli* at the class level and *Atopobium vaginae*. HPV infection without CIN or cancerous lesions (HPV group) was strongly associated with *Megasphaera*. The most abundant bacterium in the LSIL group was *Prevotella amnii*. However, *Prevotella timonensis*, *Shuttleworthia* and *Streptococcaceae* at the family level were three taxa related to HSIL. Furthermore, more taxa were associated with the Cancer group including *Bacillus*, *Sneathia*, *Acidovorax*, *Oceanobacillus profundus*, *Fusobacterium*, *Veillonellaceae* at the family level, *Anaerococcus* and *Porphyromonas uenonis*.

### Characteristics of vaginal community state types (CSTs) for different groups

**Fig. 6:**
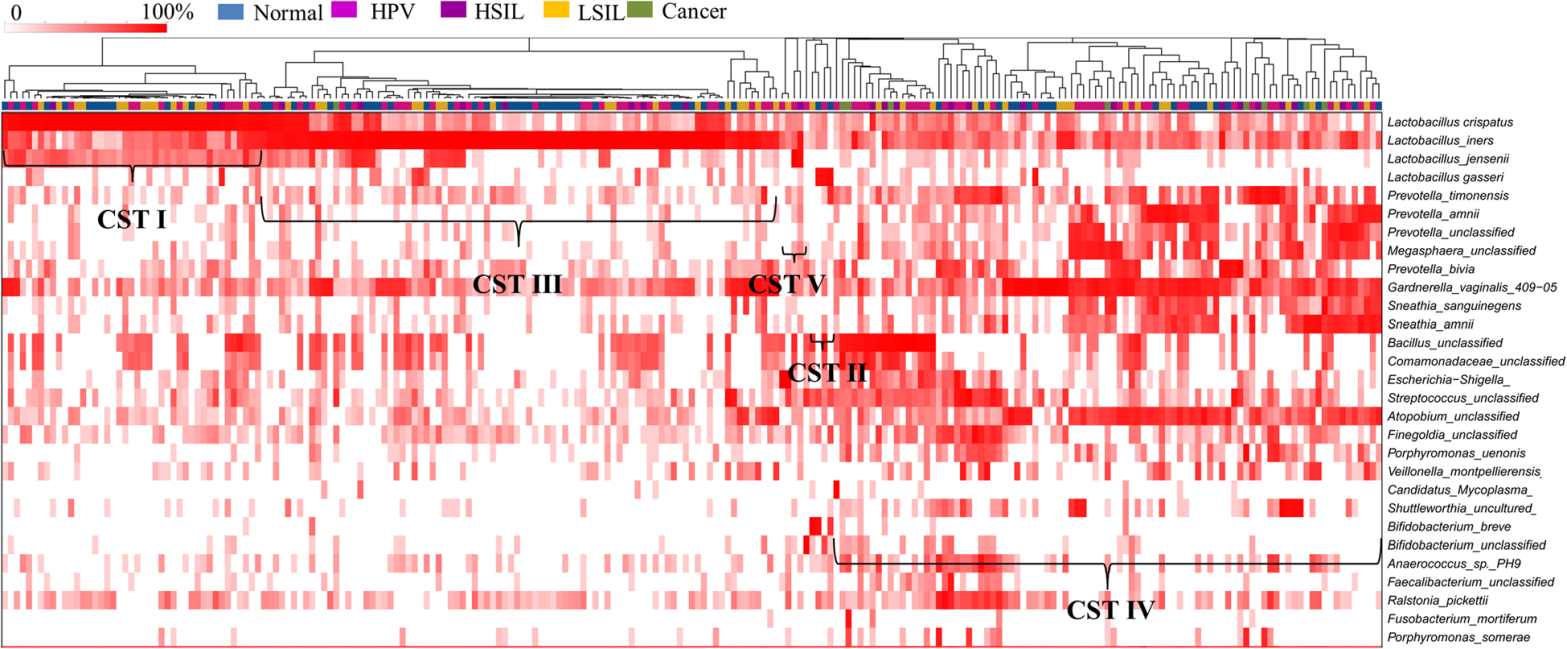
Heat map of the relative abundance of the 29 most abundant bacterial taxa found in the vaginal bacterial communities of all participants in the study. Ward linkage clustering was used to cluster samples based on their Jensen-Shannon distance. Identified CSTs are labeled as I, II, III, IV and V, according to a previous naming convention [10]

The vaginal bacterial CST analysis visualized by hierarchical clustering revealed that all samples clustered into five major groups: CST I, CST II, CSTII, CST IV and CST V (Fig. 6). The most commonly observed community was CST IV (91/229, 39.74%), followed by CST III (85/229, 37.12%), CST I (44/229, 19.21%), CST II (5/229, 2.18%) and CST V (4/229, 1.75%) as shown in Table 1. The proportions of CSTs in different groups were shown in Table 1. Samples in the Normal group were mostly assigned to CST III (32/68, 47.96%). HPV infection converted the vaginal bacterial community structure from CST III to CST IV, as all of the HPV positive groups were dominated by CST IV (HPV: 32/78, 41.03%; LSIL: 18/51, 35.29%; HSIL: 13/23, 56.52%; Cancer: 8/9, 88.89%, respectively) and presented less CST III (HPV: 28/78, 35.90%; LSIL: 17/51, 33.33%; HSIL: 7/23, 30.43%; Cancer: 1/9, 11.11%, respectively). Furthermore, the proportions of CST IV were gradually augmented with the progression of the severity of CINs (Cancer: 88.89% > HSIL: 56.52% > LSIL: 35.29%).

**Table 1:** The distribution of community state types (CSTs) in different groups

	Normal	HPV	LSIL	HSIL	Cancer	Total
	N (%)	N (%)	N (%)	N (%)	N (%)	N (%)
**CST I**	14 (20.59)	14 (17.95)	14 (27.45)	2 (8.70)	0 (0)	44 (19.21)
**CST II**	2 (2.94)	2 (2.56)	1 (1.96)	0 (0)	0 (0)	5 (2.18)
**CST III**	32 (47.06)	28 (35.90)	17 (33.33)	7 (30.43)	1 (11.11)	85 (37.12)
**CST IV**	20 (29.41)	32 (41.03)	18 (35.29)	13 (56.52)	8 (88.89)	91 (39.74)
**CST V**	0 (0)	2 (0)	1 (1.96)	1 (4.35)	0 (0)	4 (1.75)

We identified the most abundant species in each sample in Fig. 6, and the distributions of the 29 most predominant species in five groups were documented in Table 2. Seventy-eight samples (34.1%) were predominated by *Lactobacillus iners*, followed by *Lactobacillus crispatus* (48 samples, 21%), *Gardnerella vaginalis* (26 samples, 11.4%), *Bacillus unclassified* (16 samples, 7%), *Sneathia amnii* (8 samples, 3.5%) and *Prevotella amnii* (7 samples, 3.1%). HPV infection was related with the decreased abundance of *Lactobacillus iners*, *Lactobacillus crispatus*, *Gardnerella vaginalis*, *Lactobacillus gasseri*, *Anaerococcus spp.*, *Atopobium unclassified* and *Porphyromonas somerae* as compared to the Normal group (Table 2). In addition, HPV infection was associated with the increased abundance of *Bacillus unclassified*, *Escherichia Shigella*, *Megasphaera unclassified*, *Streptococcus unclassified*, *Lactobacillus jensenii*, *Sneathia sanguinegens*, *Bifidobacterium unclassified*, *Candidatus Mycoplasma*, *Comamonadaceae unclassified*, *Veillonella montpellierensis*, *Faecalibacterium unclassified*, *Finegoldia unclassified*, *Fusobacterium mortiferum*, *Porphyromonas uenonis* and *Ralstonia pickettii* (Table 2). Furthermore, the abundance of *Gradnerella vaginalis* was gradually reduced with the progression of CINs severity (Cancer 0% < HSIL 4.3% < LSIL 11.8% < HPV 12.8% < Normal 13.2% as shown in Table 2).

**Table 2:** The distribution of the 29 most predominant species in different groups

	Normal	HPV	LSIL	HSIL	Cancer	Total
	N (%)	N (%)	N (%)	N (%)	N (%)	N (%)
*Lactobacillus crispatus*	16 (23.5)	15 (19.2)	14 (27.5)	3 (13.0)	0 (0)	48 (21.0)
*Lactobacillus iners*	30 (44.1)	21 (27.0)	20 (39.2)	6 (26.1)	1 (11.1)	78 (34.1)
*Lactobacillus jensenii*	0 (0)	1 (1.3)	0 (0)	1 (4.3)	0 (0)	2 (0.9)
*Lactobacillus gasseri*	1 (1.5)	1 (1.3)	0 (0)	0 (0)	0 (0)	2 (0.9)
*Anaerococcus spp.*	1 (1.5)	0 (0)	0 (0)	0 (0)	0 (0)	1 (0.4)
*Atopobium unclassified*	1 (1.5)	0 (0)	0 (0)	0 (0)	0 (0)	1 (0.4)
*Bacillus unclassified*	0 (0)	7 (9.0)	5 (9.8)	3 (13.0)	1 (11.1)	16 (7.0)
*Bifidobacterium breve*	1 (1.5)	0 (0)	1 (2.0)	0 (0)	0 (0)	2 (0.9)
*Bifidobacterium unclassified*	0 (0)	1 (1.3)	0 (0)	0 (0)	0 (0)	1 (0.4)
*Candidatus Mycoplasma*	0 (0)	1 (1.3)	0 (0)	0 (0)	0 (0)	1 (0.4)
*Comamonadaceae unclassified*	0 (0)	0 (0)	0 (0)	0 (0)	1 (11.1)	1 (0.4)
*Veillonella montpellierensis*	0 (0)	1 (1.3)	0 (0)	0 (0)	0 (0)	1 (0.4)
*Escherichia Shigella*	0 (0)	3 (3.8)	1 (2.0)	0 (0)	0 (0)	4 (1.7)
*Faecalibacterium unclassified*	0 (0)	0 (0)	0 (0)	1 (4.3)	0 (0)	1 (0.4)
*Finegoldia unclassified*	0 (0)	1 (1.3)	0 (0)	0 (0)	0 (0)	1 (0.4)
*Fusobacterium mortiferum*	0 (0)	0 (0)	0 (0)	0 (0)	1 (11.1)	1 (0.4)
*Gardnerella vaginalis*	9 (13.2)	10 (12.8)	6 (11.8)	1 (4.3)	0 (0)	26 (11.4)
*Megasphaera unclassified*	0 (0)	1 (1.3)	0 (0)	1 (4.3)	0 (0)	2 (0.9)
*Porphyromonas somerae*	1 (1.5)	0 (0)	0 (0)	0 (0)	0 (0)	1 (0.4)
*Porphyromonas uenonis*	0 (0)	0 (0)	0 (0)	0 (0)	1 (11.1)	1 (0.4)
*Prevotella amnii*	2 (3.0)	3 (3.8)	0 (0)	1 (4.3)	1 (11.1)	7 (3.1)
*Prevotella bivia*	1 (1.5)	0 (0)	0 (0)	1 (4.3)	1 (11.1)	3 (1.3)
*Prevotella timonensis*	1 (1.5)	2 (2.6)	0 (0)	1 (4.3)	0 (0)	4 (1.7)
*Prevotella unclassified*	1 (1.5)	1 (1.3)	0 (0)	1 (4.3)	0 (0)	3 (1.3)
*Ralstonia pickettii*	0 (0)	1 (1.3)	0 (0)	0 (0)	0 (0)	1 (0.4)
*Shuttleworthia uncultured*	1 (1.5)	2 (2.6)	1 (2.0)	2 (8.7)	0 (0)	6 (2.6)
*Sneathia amnii*	2 (2.9)	3 (3.8)	2 (3.9)	0 (0)	1 (11.1)	8 (3.5)
*Sneathia sanguinegens*	0 (0)	0 (0)	0 (0)	0 (0)	1 (11.1)	1 (0.4)
*Streptococcus unclassified*	0 (0)	3 (3.8)	1 (2.0)	1 (4.3)	0 (0)	5 (2.1)

## Discussion

Our study addressed a not well-elucidated topic about the association between HPV infection and related CINs or cervical cancer and vaginal microbiome in Chinese cohorts. We observed that HPV infection increased vaginal bacterial richness and diversity regardless of the status of CINs. The vaginal bacterial richness and diversity were further augmented in the women with cervical cancer. *Lactobacillus* was the most abundant genus in all groups. HPV infection had a negative influence on the abundances of *Lactobacillus*, *Gardnerella* and *Atopobium*. Accordingly, HPV infection increased the relative abundance of *Prevotella*, *Bacillus*, *Anaerococcus*, *Sneathia*, *Megasphaera*, *Streptococcus* and *Anaerococcus*. The increased proportions of *Bacillus*, *Anaerococcus* and the reduced abundance of *Gradnerella vaginalis* were probably related with the progression of CINs severity. HPV infection without CINs or cancerous lesions was strongly associated with *Megasphaera*. The most abundant bacterium in the LSIL group was *Prevotella amnii*. However, *Prevotella timonensis*, *Shuttleworthia* and *Streptococcaceae* at the family level were three taxa related to HSIL. Furthermore, more taxa were associated with the Cancer group including *Bacillus*, *Sneathia*, *Acidovorax*, *Oceanobacillus profundus*, *Fusobacterium*, *Veillonellaceae* at the family level, *Anaerococcus* and *Porphyromonas uenonis*. Samples in the Normal group were mostly assigned to CST III. HPV infection converted the vaginal bacterial community structure from CST III to CST IV. Furthermore, the proportions of CST IV were gradually augmented with the progression of the severity of CINs.

Most of the studies have proven that HPV infection can increase vaginal bacterial richness and diversity and lower the percentage of *Lactobacillus* [4, 8, 17, 18, 23, 24, 40, 41], and our results are in agreement with these previous studies. However, a few studies found no difference between HPV positive and negative groups [25, 42]. HPV infection is thought to alter the acidic environment of the vagina, which might promote outbreaks of bacteria [24]. In addition, HPV infection might lead to changes in the vaginal microbiota by inducing host mucosal immune response and genital infalmmation [41, 43]. High genital inflammation with elevated vaginal PH and non-*Lactobacillus*-dominant VM have been associated with HPV persistence and progression to cervical cancer [44]. But the underlying biological mechanisms are still unclear. On the other hand, Mitra et al. reported that increasing CINs severity was associated with decreasing relative abundance of *Lactobacillus* and increasing bacterial diversity [26]. Differently, we only observed a higher bacterial richness and diversity in group Cancer than in groups HSIL, LSIL or HPV, but no differences were detected when compared between each two of groups HPV, LSIL and HSIL. Some other studies also found no connection between the diversity of VM and the CINs progression [45, 46]. It is notable that the study of Mitra et al. [26] did not distinguish the influence of HPV infection from precancerous or cancerous lesions on the diversity of VM.

Similarly to the previous studies [2, 4, 17, 18, 23, 24, 40, 42, 47], we found increased abundances of several anaerobic bacteria such as *Prevotella*, *Bacillus*, *Anaerococcus*, *Sneathia*, *Megasphaera*, *Streptococcus* and *Anaerococcus* in HPV-infected women. We recognized *Megasphaera* of *Firmicutes* phylum as the most significant genus related with HPV infection, while Lee et al. identified *Sneathia spp.* of *Fusobacteria* phylum as the microbiological marker of HPV infection [24]. Over all, a microbial environment with a higher proportion of anaerobic bacteria and a lower proportion of *Lactobaillus* spp. is more likely to HPV infection. A surprising finding in this study was that the proportions of *Gardnerella* and *Atopobium*, were reduced in HPV-positive women. *Gardnerella vaginalis* and *Atopobium vaginae* were thought to be associated with BV [48]. Gao et al. reported that these two taxa were more frequently detected in HPV-infected women [23]. However, the method used in their study was totally different from this research. Another study in a Caucasian cohort proposed *Atopobium spp.* and sialidase-encoding gene from *Gardnerella vaginalis* as microbial markers of HPV persistence [40]. It is hypothesized that the enzyme (sialidase) facilitates the destruction of the mucus layer on the vaginal epithelium and entraps anaerobic bacteria such as *Prevotella* and *Atopobium*. The potential reason for this different result remains to explore. To distinguish the influence of HPV infection from precancerous or cancerous lesions on vaginal microbiota, we compared the vaginal bacterial composition between group HPV and groups LSIL, HSIL or Cancer. We found that the proportions of two specific taxa, *Bacillus* and *Anaerococcus*, were positively related with the progression of CINs severity. Furthermore, we identified respective taxa for different stages of CIN lesions. Mitra et al. also reported that higher levels of *Sneathia sanguinegens*, *Anaerococcus tetradius* and *Peptostreptococcus anaerobius* were characterized in HSIL compared to LSIL [26]. Corresponding to the impact of HPV infection on the vaginal microbes, we also found that the abundance of *Gradnerella vaginalis* was gradually reduced with the progression of CINs severity. However, some other studies [19, 27] thought that an enrichment of *Gradnerella vaginalis* and *Atopobium vaginae* had a higher CIN risk.

In accordance with the results from an Asian population in the study by Ravel et al. [10], the most abundant CST in Normal group was CST III. The most dominant CST in the HPV positive groups (HPV, LSIL, HSIL and Cancer) was CST IV. We observed HPV infection to be associated with an increased proportion of CST IV, and furthermore its proportion was gradually augmented with the progression of the severity of CINs. It has also been reported by two longitudinal studies that the majority of HPV-positive samples were composed of CST IV (dominated by anaerobic bacteria), and CST IV was related with an increased risk of transitioning to an HPV-positive state [24, 49]. Mitra et al. also found that the rate of CST IV was increased 2 fold in women with LSIL, 3 fold in women with HSIL and 4 fold in women with invasive cancer [26]. CST IV is associated with higher levels of amine production, resulting in carcinogens nitrosamine production [50].

The strength of this study is that it interpreted the vaginal microbial compositions of a large cohort of Chinese women with different stages of HPV-related diseases using high throughput sequencing method, which has not yet been well elucidated. We found that HPV infection increased vaginal bacterial richness and diversity regardless of the status of CINs. The specific microbes and the vaginal bacterial structure were related with the progression of CINs severity in Chinese women. The limitations of this study were that it was a cross-sectional study. Hence, we could not conclude any causal relationship between the VM and HPV infection or CIN diseases. We have to conduct longitudinal studies to study relationships between the dynamics of the VM and the persistence or clearance of HPV infection, and the progression or remission of CIN diseases. In addition, the underlying biological mechanisms also need to be detailed.

## Conclusion

This work interpreted the differential vaginal bacteria under HPV infection and various precancerous or cancerous lesions in a Chinese cohort. We distinguished the specific microbes and the vaginal bacterial structure that were related with the progression of CINs severity in Chinese women.

## Supplementary information

**Additional file 1: Supplementary Table 1.** HPV genotyping and distribution in five groups. **Supplementary Table 2.** Characteristics of study population. **Supplementary Table 3** Sequences information. **Supplementary Table 4.** The comparison between two groups tested by ANOSIM test.

**Additional file 2: Supplementary figure 1.** Flow chart of 229 participants. **Supplementary figure 2**: Heat map of relative abundance for the 30 most abundant bacterial phyla found in the vaginal bacterial communities of 5 groups. **Supplementary figure 3.** Heat map of relative abundance for the 30 most abundant bacterial genus found in the vaginal bacterial communities of 5 groups.

## Data Availability

All reads obtained were submitted to NCBI Sequence Read Archive (SRA) under the accession number SRP122481 (https://www.ncbi.nlm.nih.gov//bioproject/PRJNA415526).

## Abbreviations

HPV: Human papillomavirus
LSIL: Low-grade squamous intraepithelial lesion
HSIL: High-grade squamous intraepithelial lesions
BV: Bacterial vaginosis
VM: Vaginal microbiome
HIV: Human immunodeficiency virus
ANOSIM: Analysis of similarities
CST: Community state type
CIN: Cervical intraepithelial neoplasia
TCT: ThinPrep cytology test
OTUs: Operational taxonomic units
RDP: Ribosomal database project
PCoA: Principal coordinates analysis
LEfSe: Linear discriminant analysis (LDA) effect size
FDR: False discovery rate
