# Supplementary material for: Human papillomavirus infection and cervical intraepithelial neoplasia progression are associated with increased vaginal microbiome diversity in a Chinese cohort
Source: BMC Infect Dis. 2020 Aug 26;20:629. doi: 10.1186/s12879-020-05324-9 (PMC7449047; doi:10.1186/s12879-020-05324-9)
Supplement: Supplementary file 1 — Additional file 1: Supplementary Table 1. HPV genotyping and distribution in five groups. Supplementary Table 2. Characteristics of study population. Supplementary Table 3 Sequences information. Supplementary Table 4. The comparison between two groups tested by ANOSIM test. [file 12879_2020_5324_MOESM1_ESM.doc]

Supplementary Material

# Human papillomavirus infection and cervical intraepithelial neoplasia progression are associated with increased vaginal microbiome diversity in a Chinese cohort

Yulian Chen^a,b,c#^, Xingdi Qiu^a,b,c#^, Wenjing Wang^a,b,c^, Dong Li^a,b,c^, Anyue Wu^a,b,c^, Zubei Hong^a,b,c^, Wen Di^a,b,c^*, Lihua Qiu^a,b,c^*

^a^Department of Gynecology and Obstetrics, Ren ji Hospital, School of Medicine, Shanghai Jiao Tong University, Shanghai, China

^b^Shanghai Key Laboratory of Gynecologic Oncology, Ren ji Hospital, School of Medicine, Shanghai Jiao Tong University, Shanghai, China

^c^State Key Laboratory of Oncogenes and Related Genes, Shanghai Cancer Institute, Ren ji Hospital, School of Medicine, Shanghai Jiao Tong University, Shanghai, China

**Correspondence:**Lihua Qiu (email: [lilyqiulh@126.com](mailto:lilyqiulh@126.com)) and Wen Di (email:diwen163@163.com)

**Supplementary Table 1: HPV genotyping and distribution in five groups**

|  | **HPV** | **LSIL** | **HSIL** | **Cancer** |
| --- | --- | --- | --- | --- |
| **HPV genotype** | **N (%)** | | | |
| 16/18 | 13 (17%) | 10 (20%) | 6 (26%) | 4 (44%) |
| Other high-risk subtypes | 0 | 25 (49%) | 10 (43%) | 1 (10%) |
| Low-risk subtypes | 65 (83%) | 16 (31%) | 7 (30%) | 4 (44%) |

**Supplementary Table 2: Characteristics of study population**

**BMI: Body mass index; Quantitative data were shown as mean ± SD, one way ANOVA was used to compare the differences among five groups; Frequency data were shown as N (%), chi-square test was used to compare the differences among five groups; P<0.05 was significant, ^***^: P≤0.001；^*^P<0.05.**

|  | **Normal**  **(N=68)** | **HPV**  **(N=78)** | **LSIL**  **(N=51)** | **HSIL**  **(N=30)** | **Cancer**  **(N=9)** | **P value** |
| --- | --- | --- | --- | --- | --- | --- |
| **Age（year）** | 43.00±8.69 | 47.78±9.63 | 46.00±10.19 | 43.70±10.74 | 56.11±9.02 | <0.001^***^ |
| **Smoking** |  |  |  |  |  | <0.001^***^ |
| **Active** | 0（0） | 1（1.3） | 0（0） | 2（8.7） | 0（0） |  |
| **Passive** | 25 (32.1) | 35 (44.9) | 16 (31.4) | 6 (26.1) | 2 (22.2) |  |
| **Never** | 53 (67.9) | 42 (53.8) | 35 (68.6) | 15 (65.2) | 7 (77.8) |  |
| **BMI (kg/m^2^)** | 22.94±2.74 | 22.76±2.97 | 22.44±2.94 | 22.85±2.70 | 23.99±0.68 | <0.001^***^ |
| **Sexual age (year)** | 23.00±2.34 | 24.00±2.96 | 24.00±3.08 | 24.00±3.13 | 28.00±3.53 | <0.001^***^ |
| **Sexual partners** | 1.00±0.24 | 1.00±0.61 | 1.00±0.51 | 1.00±0.67 | 2.00±0.71 | 0.164 |
| **Gesity** | 2.00±0.99 | 2.00±1.24 | 3.00±1.15 | 3.00±1.82 | 3.00±0.71 | 0.01^*^ |
| **Parity** | 1.00±0.50 | 1.00±0.41 | 1.00±0.75 | 1.00±0.44 | 2.00±0.71 | 0.08 |

**Supplementary Table 3: Sequences information**

|  | **Sample ID** | **Reads Number** | **OTU Number** | **Average reads per group**  **(Mean ± SD)** | **Average OTUs per group**  **(Mean ± SD)** |
| --- | --- | --- | --- | --- | --- |
| **Normal** | 4338 | 25614 | 169 | 26778.22 ± 4480.73 | 46.17 ±38.06 |
|  | 4339 | 24026 | 62 |  |  |
|  | 4340 | 22706 | 22 |  |  |
|  | 4341 | 34562 | 45 |  |  |
|  | 4343 | 37373 | 70 |  |  |
|  | 4344 | 28553 | 79 |  |  |
|  | 4346 | 30560 | 56 |  |  |
|  | 4347 | 29494 | 24 |  |  |
|  | 4348 | 23773 | 35 |  |  |
|  | 4349 | 28283 | 43 |  |  |
|  | 4350 | 37170 | 48 |  |  |
|  | 4351 | 29005 | 21 |  |  |
|  | 4352 | 26837 | 49 |  |  |
|  | 4353 | 33863 | 77 |  |  |
|  | 4354 | 23413 | 54 |  |  |
|  | 4355 | 30645 | 51 |  |  |
|  | 4357 | 23166 | 239 |  |  |
|  | 4359 | 20456 | 28 |  |  |
|  | 4360 | 21131 | 19 |  |  |
|  | 4362 | 29077 | 66 |  |  |
|  | 4363 | 24856 | 28 |  |  |
|  | 4364 | 26359 | 154 |  |  |
|  | 4365 | 24986 | 32 |  |  |
|  | 4366 | 32050 | 28 |  |  |
|  | 4367 | 26228 | 67 |  |  |
|  | 4368 | 31679 | 39 |  |  |
|  | 4369 | 32370 | 37 |  |  |
|  | 4370 | 22386 | 38 |  |  |
|  | 4374 | 21815 | 23 |  |  |
|  | 4375 | 26357 | 25 |  |  |
|  | 4380 | 29852 | 14 |  |  |
|  | 4381 | 25000 | 30 |  |  |
|  | 4382 | 30663 | 44 |  |  |
|  | 4383 | 27701 | 20 |  |  |
|  | 4384 | 21253 | 43 |  |  |
|  | 4385 | 26870 | 71 |  |  |
|  | 4387 | 28749 | 66 |  |  |
|  | 4388 | 33182 | 22 |  |  |
|  | 4389 | 30946 | 23 |  |  |
|  | 4390 | 21922 | 27 |  |  |
|  | 4391 | 29645 | 10 |  |  |
|  | 4392 | 20773 | 41 |  |  |
|  | 4393 | 23928 | 32 |  |  |
|  | 4394 | 26932 | 32 |  |  |
|  | 4395 | 20717 | 38 |  |  |
|  | 4397 | 20858 | 17 |  |  |
|  | 4398 | 31650 | 14 |  |  |
|  | 4399 | 21412 | 36 |  |  |
|  | 4401 | 28170 | 18 |  |  |
|  | 4404 | 26976 | 40 |  |  |
|  | 4406 | 21835 | 13 |  |  |
|  | 4408 | 25641 | 18 |  |  |
|  | 4409 | 34161 | 72 |  |  |
|  | 4410 | 23005 | 37 |  |  |
|  | 4411 | 29198 | 43 |  |  |
|  | 4412 | 24660 | 51 |  |  |
|  | 4414 | 25077 | 68 |  |  |
|  | 4415 | 28763 | 89 |  |  |
|  | 4416 | 31053 | 44 |  |  |
|  | 4417 | 27323 | 20 |  |  |
|  | 4418 | 23400 | 29 |  |  |
|  | 4419 | 21055 | 57 |  |  |
|  | 4420 | 21074 | 18 |  |  |
|  | 4421 | 20399 | 41 |  |  |
|  | 4422 | 21828 | 24 |  |  |
|  | 4434 | 22381 | 19 |  |  |
|  | 4471 | 32443 | 25 |  |  |
|  | 4471 | 23838 | 20 |  |  |
| **HPV** | 152_GPZ | 36647 | 782 | 29710.78 ±5725.17 | 157.31 ± 144.15 |
|  | 374_BXR | 32378 | 55 |  |  |
|  | 48_ZQ | 25572 | 160 |  |  |
|  | 39_SGH | 21231 | 725 |  |  |
|  | 16_CXH | 36411 | 132 |  |  |
|  | 359_ZXM | 34251 | 31 |  |  |
|  | 11_NX | 30974 | 249 |  |  |
|  | 46_ZJQ | 36731 | 107 |  |  |
|  | 167_GAL | 39864 | 141 |  |  |
|  | 392_XHR | 34173 | 50 |  |  |
|  | 105_HJ | 39237 | 298 |  |  |
|  | 124_WHL | 20363 | 332 |  |  |
|  | 201_WJH | 31762 | 130 |  |  |
|  | 349_XWF | 34148 | 93 |  |  |
|  | 25_JS | 20925 | 183 |  |  |
|  | 45_WP | 24491 | 78 |  |  |
|  | 162_ZRH | 25203 | 101 |  |  |
|  | 27_WJQ | 27307 | 132 |  |  |
|  | 305_SX | 20599 | 59 |  |  |
|  | 32_CHZ | 27047 | 142 |  |  |
|  | 49_WYL | 35211 | 317 |  |  |
|  | 54_WWL | 26057 | 156 |  |  |
|  | 395_QXL | 24447 | 55 |  |  |
|  | 357_JYM | 29184 | 46 |  |  |
|  | 99_LYM | 24864 | 156 |  |  |
|  | 318_LCQ | 20098 | 33 |  |  |
|  | 56_XP | 37583 | 121 |  |  |
|  | 18_WLM | 22872 | 199 |  |  |
|  | 9_LRD | 36412 | 70 |  |  |
|  | 3_YLL | 29717 | 147 |  |  |
|  | 372_THL | 32303 | 35 |  |  |
|  | 367_GJL | 28380 | 80 |  |  |
|  | 288_ZLX | 35946 | 44 |  |  |
|  | 73_JZY | 28294 | 131 |  |  |
|  | 81_DHG | 28425 | 156 |  |  |
|  | 72_GXM | 39047 | 67 |  |  |
|  | 196_LH | 34105 | 94 |  |  |
|  | 397_HJF | 20746 | 289 |  |  |
|  | 65_XZY | 31333 | 171 |  |  |
|  | 57_HXY | 38519 | 216 |  |  |
|  | 315_LXH | 33029 | 22 |  |  |
|  | 145_WWH | 22627 | 71 |  |  |
|  | 123_CGY | 32789 | 205 |  |  |
|  | 301_LJ | 21292 | 49 |  |  |
|  | 285_CWH | 34224 | 17 |  |  |
|  | 268_XYD | 24652 | 38 |  |  |
|  | 128_SYX | 31641 | 138 |  |  |
|  | 34_CFM | 34358 | 409 |  |  |
|  | 173_QYP | 25690 | 118 |  |  |
|  | 134_WMZ | 33247 | 210 |  |  |
|  | 387_WJX | 24160 | 70 |  |  |
|  | 360_TCJ | 34348 | 34 |  |  |
|  | 114_QJF | 22881 | 160 |  |  |
|  | 76_LLH | 27157 | 266 |  |  |
|  | 78_ZHY | 20457 | 69 |  |  |
|  | 154_XHM | 29429 | 74 |  |  |
|  | 141_XY | 31753 | 133 |  |  |
|  | 17_CWL | 23761 | 324 |  |  |
|  | 356_LMZ | 23755 | 128 |  |  |
|  | 35_QR | 33164 | 716 |  |  |
|  | 400_LXY | 38454 | 41 |  |  |
|  | 29_OYL | 30201 | 177 |  |  |
|  | 140_HXH | 25640 | 82 |  |  |
|  | 20_TWJ | 23704 | 95 |  |  |
|  | 321_ZJM | 32544 | 163 |  |  |
|  | 14_YXH | 39555 | 105 |  |  |
|  | 51_LLD | 35070 | 124 |  |  |
|  | 180_DJL | 30710 | 132 |  |  |
|  | 347_XY | 31230 | 42 |  |  |
|  | 121_THM | 31262 | 322 |  |  |
|  | 325_XXM | 26198 | 187 |  |  |
|  | 63_WHX | 32170 | 151 |  |  |
|  | 24_LXL | 32939 | 88 |  |  |
|  | 12_GXR | 20685 | 242 |  |  |
|  | 58_TXR | 30234 | 98 |  |  |
|  | 79_XH | 26919 | 131 |  |  |
|  | 200_CHY | 39883 | 160 |  |  |
|  | 61_ZYX | 22772 | 116 |  |  |
| **LSIL** | 118_CL | 26828 | 109 | 28537.30 ± 5253.89 | 144.60 ±143.29 |
|  | 13_SQ | 33367 | 365 |  |  |
|  | 157_ZR | 28995 | 104 |  |  |
|  | 159_FBH | 38417 | 167 |  |  |
|  | 160_QYF | 27639 | 540 |  |  |
|  | 22_OGL | 21435 | 337 |  |  |
|  | 227_JYM | 31691 | 32 |  |  |
|  | 231_WJP | 34789 | 63 |  |  |
|  | 238_SYF | 22791 | 86 |  |  |
|  | 251_ZFD | 35236 | 122 |  |  |
|  | 252_CAL | 21610 | 31 |  |  |
|  | 258_CYP | 29995 | 99 |  |  |
|  | 26_JLW | 34898 | 144 |  |  |
|  | 266_ZJH | 29664 | 37 |  |  |
|  | 269_DLQ | 24386 | 27 |  |  |
|  | 270_TD | 26699 | 59 |  |  |
|  | 275_WRF | 33771 | 75 |  |  |
|  | 28_LFH | 35442 | 407 |  |  |
|  | 284_SMH | 24648 | 55 |  |  |
|  | 290_XZ | 23392 | 53 |  |  |
|  | 30_WT | 33985 | 116 |  |  |
|  | 309_ZCF | 25717 | 34 |  |  |
|  | 31_SMH | 32180 | 154 |  |  |
|  | 316_BPL | 36170 | 73 |  |  |
|  | 322_WCH | 30438 | 33 |  |  |
|  | 341_FXF | 27016 | 72 |  |  |
|  | 344_WQZ | 24789 | 117 |  |  |
|  | 345_WXC | 38126 | 36 |  |  |
|  | 348_GXF | 21625 | 102 |  |  |
|  | 36_SLF | 29149 | 759 |  |  |
|  | 361_YPQ | 25179 | 41 |  |  |
|  | 37_YYM | 32904 | 97 |  |  |
|  | 371_MJ | 35680 | 32 |  |  |
|  | 383_FLJ | 24896 | 215 |  |  |
|  | 384_JKM | 23593 | 36 |  |  |
|  | 385_ZLQ | 22520 | 21 |  |  |
|  | 386_CLH | 33654 | 48 |  |  |
|  | 43_ZLY | 31719 | 95 |  |  |
|  | 44_DYH | 23567 | 121 |  |  |
|  | 52_CYH | 33910 | 120 |  |  |
|  | 53_SXH | 20159 | 177 |  |  |
|  | 59_ZQ | 20863 | 258 |  |  |
|  | 66_XF | 23135 | 180 |  |  |
|  | 75_LMS | 27398 | 173 |  |  |
|  | 77_HQ | 24284 | 106 |  |  |
|  | 80_FL | 22316 | 172 |  |  |
|  | 83_DLY | 36718 | 216 |  |  |
|  | DMH | 24607 | 384 |  |  |
|  | YQM | 25755 | 66 |  |  |
|  | YXX | 29120 | 264 |  |  |
|  | HMJ | 34571 | 132 |  |  |
| **HSIL** | 390_GHM | 30761 | 28 | 29685.50 ± 4754.64 | 116.05 ±112.47 |
|  | 355_HJH | 23122 | 110 |  |  |
|  | 331_QY | 28480 | 32 |  |  |
|  | 74_WJJ | 25156 | 285 |  |  |
|  | 376_GXH | 30001 | 32 |  |  |
|  | 358_SHQ | 27742 | 69 |  |  |
|  | 350_CLJ | 34617 | 52 |  |  |
|  | 389_CHL | 35876 | 55 |  |  |
|  | 15_CHQR | 35256 | 414 |  |  |
|  | 271_LL | 33627 | 46 |  |  |
|  | 178_XSS | 29757 | 202 |  |  |
|  | 343_XHQ | 24955 | 36 |  |  |
|  | 10_ZYM | 37988 | 317 |  |  |
|  | 216_LHY | 34460 | 266 |  |  |
|  | 116_SMH | 34322 | 193 |  |  |
|  | 171_WY | 31554 | 45 |  |  |
|  | 398_HNF | 22186 | 51 |  |  |
|  | 335_YYP | 32048 | 40 |  |  |
|  | 323_HHX | 27974 | 40 |  |  |
|  | 336_XCL | 25191 | 65 |  |  |
|  | 399_WSX | 32372 | 51 |  |  |
|  | 286_LX | 25853 | 44 |  |  |
|  | 50_TXF | 22155 | 131 |  |  |
| **Cancer** | 67_WMY | 30744 | 306 | 33995.7± 5473.90 | 256.70 ±174.78 |
|  | 23_CY | 39752 | 210 |  |  |
|  | 33_WFF | 31295 | 322 |  |  |
|  | 55_TLL | 25638 | 599 |  |  |
|  | 106_XGX | 36139 | 112 |  |  |
|  | 380_HGD | 37890 | 88 |  |  |
|  | 126_YYD | 26405 | 397 |  |  |
|  | 377_SQQ | 39836 | 105 |  |  |
|  | 64_WR | 39886 | 377 |  |  |

**Supplementary Table 4: The comparison between two groups tested by ANOSIM test**

| Group | R value | P value |
| --- | --- | --- |
| Normal vs, HPV | 0.2917 | 0.001 |
| Normal vs. LSIL | 0.1279 | 0.001 |
| Normal vs. HSIL | 0.0732 | 0.07 |
| Normal vs. Cancer | 0.284 | 0.001 |
| HPV vs. LSIL | 0.0484 | 0.019 |
| HPV vs. HSIL | 0.1375 | 0.001 |
| HPV vs. Cancer | -0.0359 | 0.656 |
| LSIL vs. HSIL | 0.0072 | 0.37 |
| LSIL vs. Cancer | -0.0135 | 0.618 |
| HSIL vs. Cancer | 0.068 | 0.088 |
