# Supplementary material for: Human papillomavirus infection and cervical intraepithelial neoplasia progression are associated with increased vaginal microbiome diversity in a Chinese cohort
Source: BMC Infect Dis. 2020 Aug 26;20:629. doi: 10.1186/s12879-020-05324-9 (PMC7449047; doi:10.1186/s12879-020-05324-9)
Supplement: Supplementary file 2 — Additional file 2: Supplementary figure 1. Flow chart of 229 participants. Supplementary figure 2: Heat map of relative abundance for the 30 most abundant bacterial phyla found in the vaginal bacterial communities of 5 groups. Supplementary figure 3. Heat map of relative abundance for the 30 most abundant bacterial genus found in the vaginal bacterial communities of 5 groups. [file 12879_2020_5324_MOESM2_ESM.doc]

Supplementary Material

# Human papillomavirus infection and cervical intraepithelial neoplasia progression are associated with increased vaginal microbiome diversity in a Chinese cohort

Yulian Chen^a,b,c#^, Xingdi Qiu^a,b,c#^, Wenjing Wang^a,b,c^, Dong Li^a,b,c^, Anyue Wu^a,b,c^, Zubei Hong^a,b,c^, Wen Di^a,b,c^*, Lihua Qiu^a,b,c^*

^a^Department of Gynecology and Obstetrics, Ren ji Hospital, School of Medicine, Shanghai Jiao Tong University, Shanghai, China

^b^Shanghai Key Laboratory of Gynecologic Oncology, Ren ji Hospital, School of Medicine, Shanghai Jiao Tong University, Shanghai, China

^c^State Key Laboratory of Oncogenes and Related Genes, Shanghai Cancer Institute, Ren ji Hospital, School of Medicine, Shanghai Jiao Tong University, Shanghai, China

**Correspondence:**Lihua Qiu (email: [lilyqiulh@126.com](mailto:lilyqiulh@126.com)) and Wen Di (email:diwen163@163.com)

**Supplementary figure 1: Flow chart of 229 participants**

**
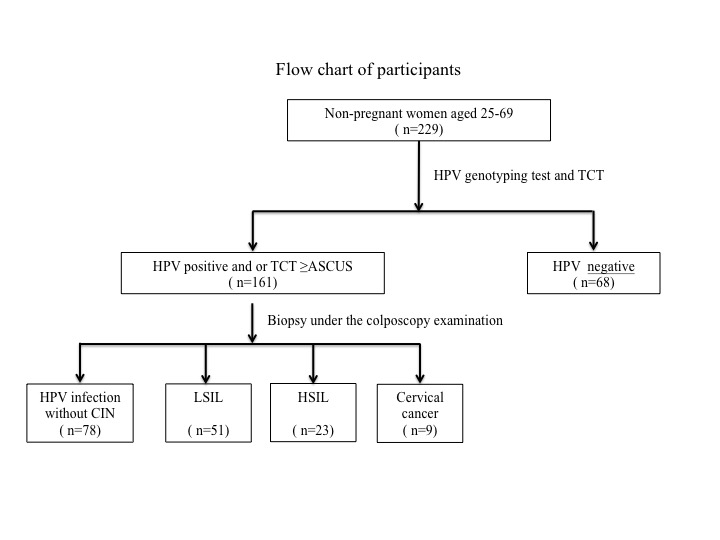
**

# Supplementary Figure 2: Heat map of relative abundance for the 30 most abundant bacterial phyla found in the vaginal bacterial communities of 5 groups


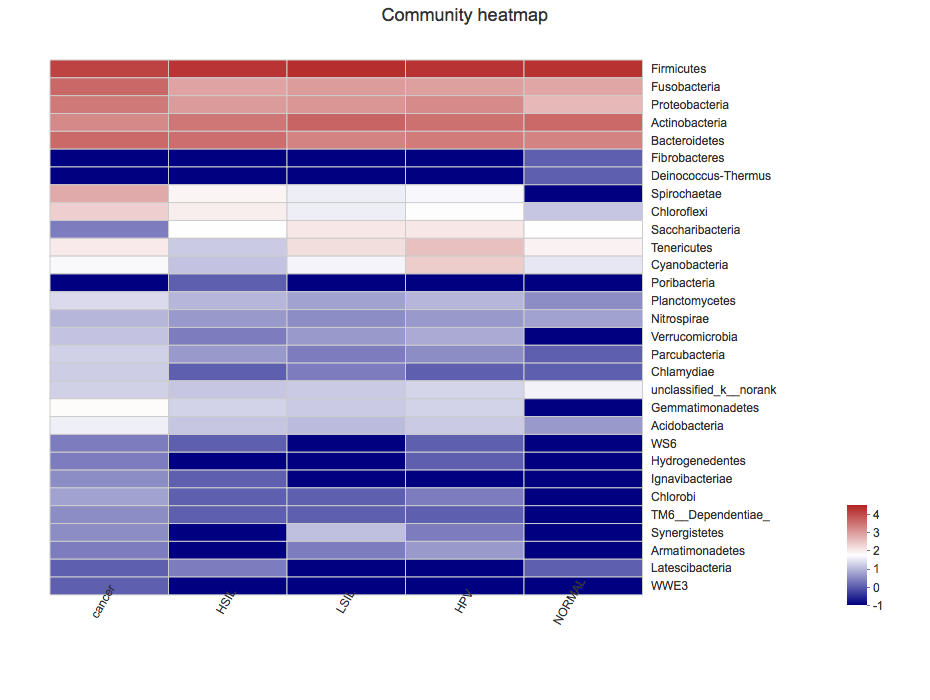


**Supplementary figure 3: Heat map of relative abundance for the 30 most abundant bacterial genus found in the vaginal bacterial communities of 5 groups**

**
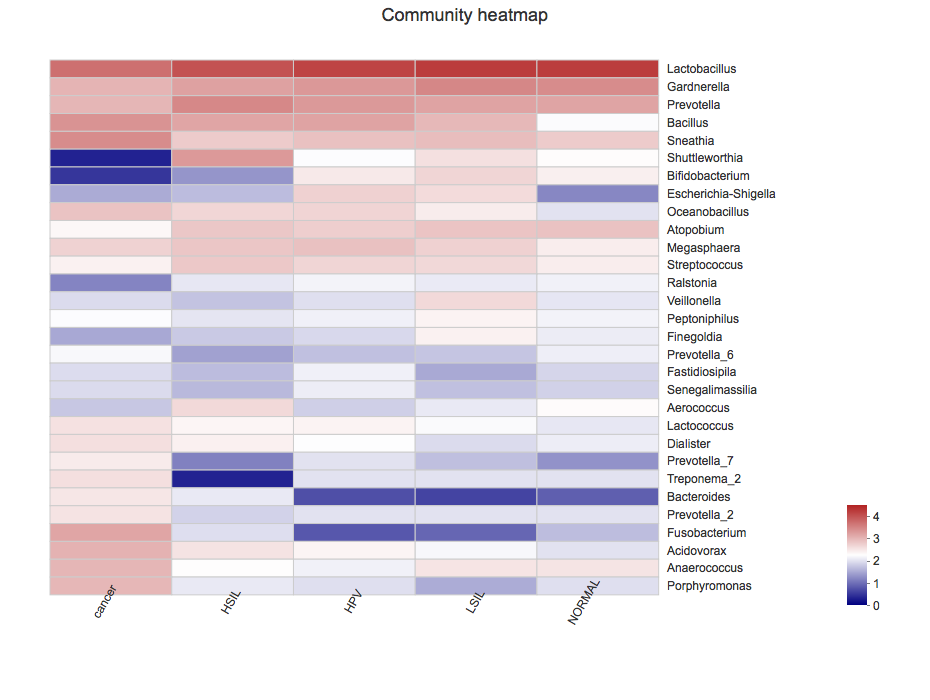
**
